# Role of Intracoronary Adrenaline in the Treatment of No-Reflow Phenomenon in Patients Undergoing Percutaneous Coronary Intervention

**DOI:** 10.7759/cureus.60338

**Published:** 2024-05-15

**Authors:** Leonard Simoni, Armand Gjana, Kristi Ziu, Alban Dibra, Artan Goda

**Affiliations:** 1 Cardiovascular Disease, University Hospital Center Mother Teresa, Tirana, ALB; 2 Cardiovascular Medicine, University Hospital Center Mother Teresa, Tirana, ALB

**Keywords:** percutaneous coronary intervention, first-line treatment, flow restoration, intracoronary adrenaline, no-reflow phenomenon

## Abstract

The no-reflow phenomenon is defined as the failure to restore coronary flow demonstrated by the reduced or missing flow in angiography despite the patent artery. There are pharmacological strategies proposed and studied to manage the no-reflow phenomenon. The medication groups used are purine nucleoside (adenosine), calcium channel blockers (verapamil, nicardipine), beta 2 receptor agonists (adrenaline, nitroprusside), fibrinolytic agents (streptokinase, tissue plasminogen activators), glycoprotein IIb/IIIa inhibitors (abciximab, tirofiban). We present a case of a woman hospitalized in non-ST elevation myocardial infarction (NSTEMI) conditions. The patient underwent coronary angiography, in which a single vessel coronary artery disease (CAD); left anterior descending (LAD) stenosis of 90% was found. In this condition, the patient underwent percutaneous coronary intervention (PCI) of LAD. The no-reflow phenomenon occurred with thrombolysis in myocardial infarction (TIMI) flow grade of 0 during the procedure.

As a consequence, the patient presented chest pain and important hypotension (BP of 70/45). Because of the hypotensive state of the patient, we decided to administer intracoronary (IC) adrenaline directly. In our case, we used adrenaline as a first-line treatment for the no-flow phenomenon due to the hypotensive state during the PCI procedure. Generally, we initially use IC nitrate or IC adenosine to resolve the phenomenon, and when the no-reflow persists we use IC adrenaline because of its side effects mentioned above. Anyway, we believe that in specific cases of hypotension and bradycardia, the use of adrenaline as the first line of therapy should be considered.

## Introduction

Percutaneous coronary intervention (PCI) is the main reperfusion strategy for acute coronary syndromes (ACS) including ST elevation myocardial Infarction (STEMI) and non-ST elevation acute coronary syndromes (NSTEACS) patients [1].

PCI is more effective and secure in restoring coronary artery patency and myocardial reperfusion than thrombolysis [2,3]. Despite all advances in PCI-related techniques and materials (guides, balloons, stents) and therapy (antithrombotic, antilipemic drugs) and good results in opening arteries, and stent positioning, normal myocardial perfusion is not always observed and considered as the no-reflow phenomenon.

The no-reflow phenomenon is defined as the failure to restore coronary flow demonstrated by the reduced or missing flow in angiography despite the patent artery. The frequency rate of no-reflow is reported up to 5% in elective procedures, and up to 60% in primary procedures [4,5]. The existence of this phenomenon is associated with worse outcomes mostly in STEMI patients [6].

Distal embolism, endothelial dysfunction, and reperfusion injury are included in the pathophysiological mechanisms of no-reflow. Different risk factors influence the presence of no-reflow phenomenon including diabetes mellitus type 2, dyslipidemia, arterial hypertension, smoking, inflammation, impaired renal function, late acute myocardial infarction (AMI) presentation, and revascularization [4]. Different factors related to revascularization procedures can prevent the no-flow phenomenon such as direct stenting, no high-pressure stenting, and thrombectomy [4].

There are pharmacological strategies proposed and studied to manage the no-reflow phenomenon. The medication groups used are purine nucleoside (adenosine), calcium channel blockers (verapamil, nicardipine), beta 2 (b2) receptor agonists (adrenaline, nitroprusside), fibrinolytic agents (streptokinase, tissue plasminogen activators), glycoprotein IIb/IIIa inhibitors (abciximab, tirofiban) [4].

So, various studies support the use of adenosine in cases of no-reflow with a dosage of 100-200 μg [7,8]. The main action of adenosine is vasodilatory but it is used also for the antiplatelet and antithrombotic effects [7,8]. Other studies support calcium channel blockers [9,10]. A meta-analysis conducted by Wang et al. studied the short-term effects of verapamil and diltiazem demonstrating the effectiveness in the treatment of the no-reflow phenomenon but with the costs of the bradycardic effect [9]. Huang et al. on the other side studied nicardipine, with an important restoration of coronary flow, and without any bradycardic effects [10]. The use of nitroprusside of sodium in no-reflow is confirmed by two meta-analyses causing vasodilatation and myocardial hyperemia [11,12]. The dosage of intracoronary sodium nitroprusside varies from 50 to 300 μg.

We present a case in which we used intracoronary adrenaline to resolve the no-reflow phenomenon, highlighting the importance of this strategy as a first-line treatment in practical clinics.

## Case presentation

**Figure 1:**
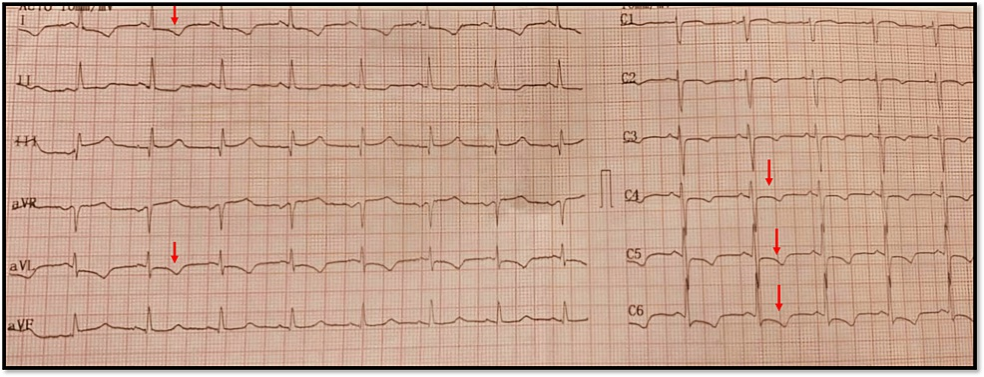
Patient electrocardiogram on admission Arrow showing negative T wave in V2-V6, D1, and aVL leads

We present a case of a 63-year-old woman hospitalized in our center complaining of chest pain with typical anginal elements accompanied by dyspnea on minimal physical effort. The patient had a three to four days history with these complaints which progressively worsened. In admission electrocardiogram (ECG) sinus rhythm and frequency 80 beats/min with negative T wave in V2-V6, D1, and an augmented vector left (aVL) lead were found (Figure 1) and high levels of troponin I of 0.645 ng/dl (<0.016ng/dl); confirming the admission diagnosis of non-ST elevation myocardial infarction (NSTEMI). Treatment with enoxaparin and clopidogrel was initiated, meanwhile, the patient was under treatment with acetylsalicylic acid, atorvastatin, and bisoprolol. The patient is known to have diabetes mellitus type 2 under oral antidiabetic therapy. 

On objective examination, the patient was oriented in time and space with the skin and mucous membranes colored. Rhythmic tones and systolic murmur at the apex were found in cardiac auscultation. In the pulmonary auscultation, rare crepitant rales in both bases were found. The abdomen is soft, and not tender on palpation. The peripheral pulses were present bilaterally. Blood pressure 120/80mmHg, heart rate = 78/min, and oxygen saturation (SpO2) = 96%. The laboratory findings are presented in Table 1.

**Table 1:** Laboratory findings on admission WBC: white blood cells, RBC: red blood cells, HB: hemoglobin, HCT: hematocrit, PLT: platelets, Tot. bilirubin: total bilirubin, AST: aspartate aminotransferase, ALT: alanine transaminase, CK: creatine kinase, CK-MB: creatine kinase-myoglobin binding, NTproBNP: N-terminal pro-b-type natriuretic peptide, HDL: high-density lipoprotein, LDL: low-density lipoprotein

Complete blood count			
Parameters	Reference range	Units	Patient’s values
RBC	4-5.6	X 10^6^/uL	4.58
HCT	37-46	%	40.1
HB	12.1-15.9	g/dL	12.8
WBC	4-10.5	K/uL	9.6
PLT	150-400	K/uL	323
Biochemistry panel			
Urea	21-43	mg/dL	31.9
Creatinine	0.57-1.11	mg/dL	0.81
Natrium	136-145	mmol/L	140
Kalium	3.5-5.1	mmol/L	3.8
Chlorine	98-107	mmol/L	102
Tot. bilirubin	0.3-1.2	mg/dL	0.76
ALT	<55	U/L	26
AST	5-34	U/L	25
CK	29-168	U/L	145
CK-MB	<3.1	ng/mL	4.9
Troponin-I	<0.016	ng/mL	0.645
NTproBNP	<125	pg/mL	416.3
Glucose	82-115	mg/dL	329
Cholesterol	<150	mg/dl	123
HDL	>40	mg/dl	38
LDL	<110	mg/dl	73
Triglyceride	<150	mg/dl	147

**Figure 2:**
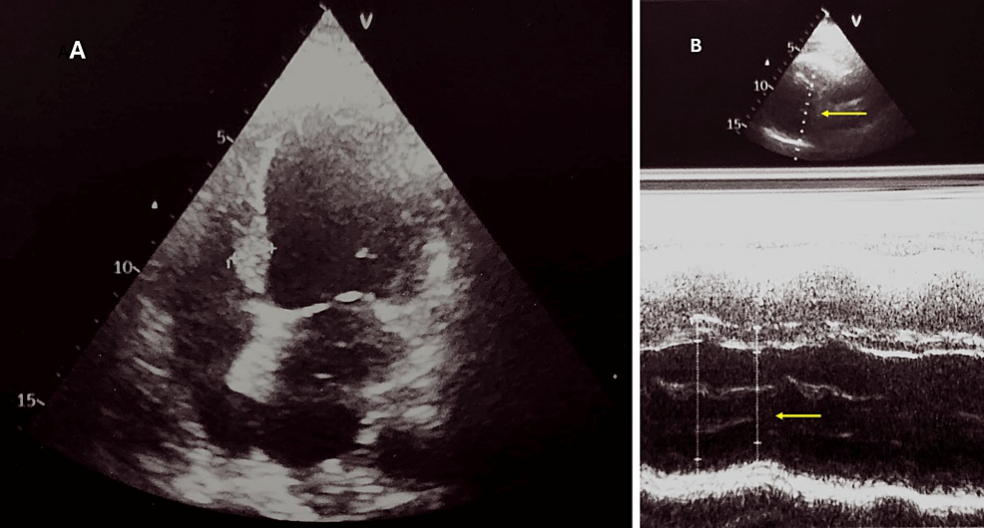
Transthoracic echocardiography (A) Apical four-chamber echo view, and (B) parasternal long axis view showing measurements (arrows) of interventricular septal diameter, end-diastolic left ventricular diameter, end-systolic ventricular diameter, and left ventricular posterior wall diameter

The transthoracic echocardiography revealed medioapical segmental hypokinesis of the anterior wall and the apex. A normal-sized left ventricle with a slightly decreased ejection fraction (EF=0.56) was found (Figure 2).

**Figure 3:**
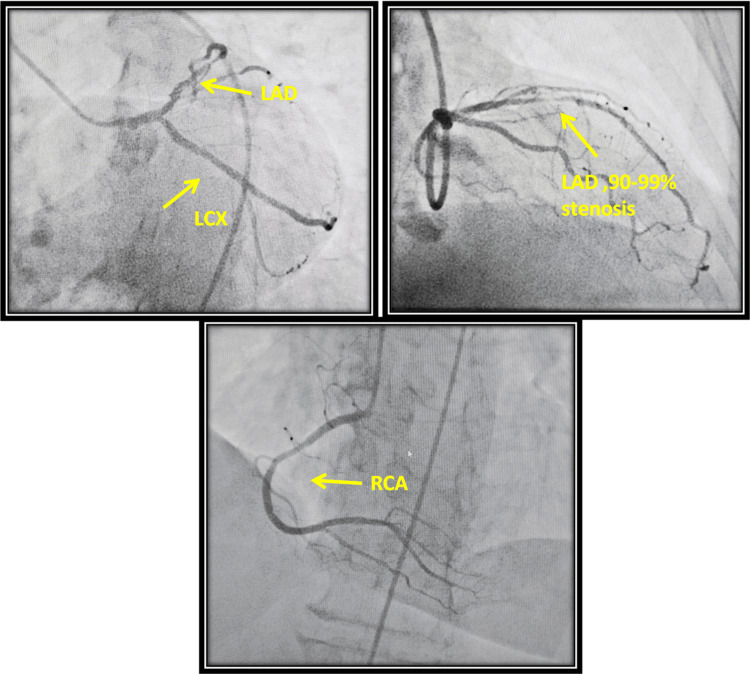
Coronary artery angiography showing LAD stenosis of 90% LAD: left anterior descending, LCX: left circumflex, RCA: right coronary artery

On the other day of admission, the patient underwent coronary angiography, in which a single vessel coronary artery disease (CAD); left anterior descending (LAD) stenosis of 90% was found, as presented in Figure 3.

**Figure 4:**
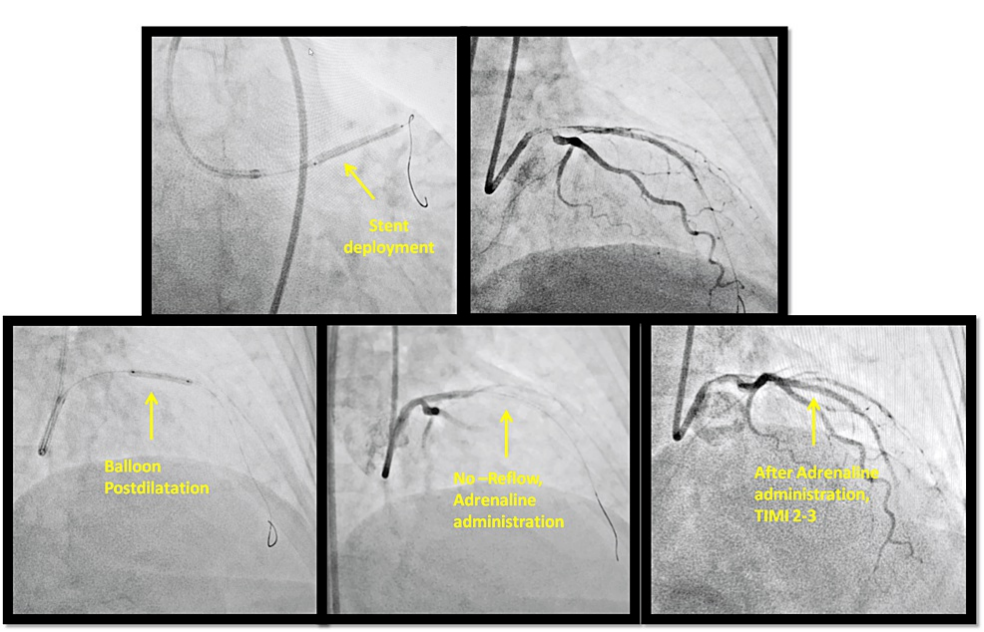
Percutaneous coronary intervention (PCI) procedure After stent deployment and balloon dilatation, a no-reflow phenomenon occurred, resolved through intracoronary (IC) adrenaline TIMI: thrombolysis in myocardial infarction

In this condition, the patient underwent PCI of LAD. After passing the guidewire beyond the stenosis in the LAD, a direct drug-eluting stent (DES), (Supraflex Cruz, Sahajanand Medical Technologies, Surat, India; 3.0x32 mm) was implanted at the stenosis level. A stent post-dilatation with a non-compliant balloon (NC Quantum Apex, Boston Scientific Corporation, Marlborough, MA; 3.0x20 mm) reached the best opening artery diameter (Figure 4). 

After the balloon use, the no-reflow phenomenon occurred with thrombolysis in myocardial infarction (TIMI) flow grade of 0. As a consequence, the patient presented chest pain and important hypotension (BP of 70/45). Because of the hypotensive state of the patient, we decided to administer IC adrenaline directly. The administration was using 1 mg ampoule of adrenaline 1 mg/1 ml. An amount of 1 ml of the ampoule was diluted in 10 ml physiologic solution and 1 ml was taken from it, in another syringe compounding 100 mcg of adrenaline. After the adrenaline use, restoration of LAD flow was observed (TIMI flow grade II-III) (Figure 4).

Two days after the PCI procedure, the patient was discharged free of symptoms and without changes in examinations related to ischemic heart disease.

## Discussion

In this report, we demonstrate a case of no-reflow management with intracoronary adrenaline in a patient with NSTEMI undergone early primary PCI. We used 100 mcg of intracoronary adrenaline. The no-reflow phenomenon can be resolved through coronary vasodilatation caused by the IC adrenaline activation of b2 receptors. Coronary arteries have a- and b-adrenergic receptors, large arteries are dominated by a1, and small coronary arterioles are dominated by b2 receptors [13]. At high doses, adrenaline can cause coronary spasm via a1 receptors, but through activation of b2 receptors at lower intracoronary doses of adrenaline, on the contrary, can cause vasodilation [14]. In addition, activating b1 receptors increases inotropic and chronotropic stimulation of the myocardium [14].

Up to now, anyway, there is no validated protocol for intracoronary adrenaline usage to restore the flow during the no-reflow phenomenon. In the literature, different case reports, case series, and various cohort and randomized studies related to using IC epinephrine as a first-line treatment or for refractory no-reflow phenomenon [15-20]. 

One of the first case series of 29 patients with refractory no-reflow during elective and primary PCI who were administered IC epinephrine to resolve the phenomenon, was presented by Skelding et al. in 2002 [15]. The administration of intracoronary epinephrine with a mean dose, of 139 ± 189 microgr significantly improved coronary flow (TIMI 3 in 69% of patients). An acceptable side effect was the increased heart rate during the administration.

Another case series of 12 patients was presented by Aksu et al. in 2015 [16]. Also in this research, the coronary flow was improved, with TIMI 3 in 75% of patients. No important adverse hemodynamic or heart rate increases were observed.

Cohort studies were conducted during the last years to compare the safety and efficacy of IC adrenaline with other treatments (IC nitrate, thrombectomy, glycoprotein IIb/IIIa inhibitors, adenosine) in the refractory no-reflow phenomenon [17,18].

Navarese et al. in 2021 studied the efficacy and safety of IC epinephrine versus the conventional treatment in STEMI patients with refractory no-reflow during primary PCI [17]. It included 30 patients with severe no-reflow after the initial failure of other treatments (IC nitrate, thrombectomy, glycoprotein IIb/IIIa inhibitors, adenosine). Administration of IC epinephrine resulted in a lower rate of sustained no-reflow, higher reperfusion rate flow, and lower rates of composite death and heart failure.

The research conducted by Darwish et al. in 2022 studied the efficacy and safety of IC adrenaline compared to adenosine for the treatment of the no-reflow phenomenon during primary PCI [18]. No-reflow was resolved more effectively and with lower rates of major adverse cardiac events in the IC epinephrine than in the IC adenosine group.

Lately, different studies have used IC adrenaline as a first-line treatment of the no-reflow phenomenon [19,20]. Hafez et al., in their 2021 study conducted in Egypt, compared the efficacy of IC verapamil and IC epinephrine in the treatment of coronary no-reflow during primary percutaneous coronary intervention (PPCI) for STEMI patients versus standard therapy (glycoprotein IIb/IIIa inhibitors) [19]. Contrary to other studies, IC verapamil resulted in a better PPCI no-reflow reversal than IC epinephrine (p=0.016) and tirofiban alone. Meanwhile, in the 2022 research of Khan et al., IC epinephrine resulted in more effective than adenosine for PPCI no-reflow phenomenon as a first in STEMI patients (TFG = 3 90% vs 78%, respectively), and similar safety (in hospital and short-term major adverse cardiac events (MACEs) [20].

On the other side, it is also important to emphasize the side effects of IC adrenaline administration. The administration of IC adrenaline, if not administered directly inside the coronary artery through microcatheters or, the distal part of the over-the-wire balloon, can increase inotropic and chronotropic stimulation of the myocardium through the activation of the b1 receptors [14]. Such stimulation can induce ventricular storm resulting in ventricular life-threatening arrhythmias.

In our case, we used adrenaline as a first-line treatment for the no-flow phenomenon due to the hypotensive state during the PCI procedure. Generally, we use IC nitrate or IC adenosine initially, to resolve the phenomenon, and when the no-reflow persists we use IC adrenaline because of its side effects mentioned above. Anyway, we believe that in specific cases of hypotension and bradycardia, the use of adrenaline as the first line of therapy should be considered.

## Conclusions

The use of adrenaline in refractory no-reflow or as a first-line therapy in specific cases of hypotension and bradycardia, during percutaneous coronary intervention (PCI) for acute coronary syndromes should be considered safe and effective. Intracoronary (IC) adrenaline acts mainly through two main mechanisms to resolve the no-reflow, firstly through b2 receptors in coronary arterioles causing vasodilatation and through the activation of cardiac b1 receptors increases inotropic and chronotropic stimulation of the myocardium, increasing the coronary flow in hypotension and bradycardia settings. Further studies are needed to confirm the effects, the dosage, and the protocol of IC adrenaline use in the no-reflow phenomenon.
